# Healthcare workers’ heterogeneous mental-health responses to prolonging COVID-19 pandemic: a full year of monthly follow up in Finland

**DOI:** 10.1186/s12888-022-04389-x

**Published:** 2022-11-19

**Authors:** Tom Rosenström, Katinka Tuisku, Jaana Suvisaari, Eero Pukkala, Kristiina Junttila, Henna Haravuori, Marko Elovainio, Toni Haapa, Pekka Jylhä, Tanja Laukkala

**Affiliations:** 1 Department of Psychology and Logopedics, Faculty of Medicine, University of Helsinki, 00014 Helsinki, Finland; 2 Department of Psychiatry, University of Helsinki and Acute Psychiatry and Consultations, HUS Helsinki University Hospital, 00029 Helsinki, Finland; 3 Finnish Institute for Health and Welfare, Mental Health Team, 00271 Helsinki, Finland; 4 Faculty of Social Sciences, Tampere University, 33100 Tampere, Finland; 5 Nursing Research Center, HUS Helsinki University Hospital and University of Helsinki, 00029 Helsinki, Finland; 6 Finnish Institute for Health and Welfare, Health Services Research, 00271 Helsinki, Finland

**Keywords:** SARS-CoV-2, Psychological distress, Sleep problems, Insomnia, Latent-class mixed models, Traumatic events

## Abstract

**Background:**

The COVID-19 pandemic strained healthcare workers but the individual challenges varied in relation to actual work and changes in work. We investigated changes in healthcare workers’ mental health under prolonging COVID-19 pandemic conditions, and heterogeneity in the mental-health trajectories.

**Methods:**

A monthly survey over a full year was conducted for employees of the HUS Helsinki University Hospital (*n* = 4804) between 4th June 2020 to 28th May 2021. Pandemic-related potentially traumatic events (PTEs), work characteristics (e.g., contact to COVID-19 patients), local COVID-19 incidence, and demographic covariates were used to predict Mental Health Index-5 (MHI-5) and Insomnia Severity Index (ISI) in generalized multilevel and latent-class mixed model regressions.

**Results:**

Local COVID-19 log-incidence (odds ratio, OR = 1.21, with 95% CI = 1.10–1.60), directly caring for COVID-19 patients (OR = 1.33, CI = 1.10–1.60) and PTEs (OR = 4.57, CI = 3.85–5.43) were all independently associated with psychological distress, when (additionally) adjusting for age, sex, profession, and calendar time. Effects of COVID-19 incidence on mental health were dissociable from calendar time (i.e., evolved in time) whereas those on sleep were not. Latent mental-health trajectories were characterized by a large class of “stable mental health” (62% of employees) and minority classes for “early shock, improving” (14%) and “early resilience, deteriorating” mental health (24%). The minority classes, especially “early shock, improving”, were more likely to live alone and be exposed to PTEs than the others.

**Conclusions:**

Healthcare workers faced changing and heterogeneous mental-health challenges as the COVID-19 pandemic prolonged. Adversity and mental ill-being may have accumulated in some employees, and factors like living arrangements may have played a role. Knowledge on employees’ demographic and socioeconomic background, as well as further research on the factors affecting employees’ resilience, may help in maintaining healthy and efficient workforce in the face of a prolonging pandemic.

**Supplementary Information:**

The online version contains supplementary material available at 10.1186/s12888-022-04389-x.

## Background

The COVID-19 pandemic represents a global threat to mental health. In the general population and among healthcare personnel, that threat has manifested in relatively high rates of symptoms of anxiety, depression, psychological distress, and post-traumatic stress disorder [1–5]. In the beginning of the COVID-19 pandemic, healthcare workers had higher rates of insomnia compared to the general population [4, 5]. Transient insomnia is an early marker of stress that represents a common response to environmental and psychological challenges. Prolonged and frequent insomnia symptoms predict higher rates of sickness absence due to psychiatric disorders, and mild insomnia symptoms were reported by 53% irrespective of profession during the COVID-19 pandemic [6–8]. Healthcare personnel’s psychological distress and sickness absence has varied by pandemic phase and profession [1, 9]. Therefore, we examine here changes in and correlates of healthcare workers’ mental health and sleep problems over time. Although general psychological distress was our primary outcome, we considered insomnia an important secondary outcome that both reflects risk of reactive distress and has locally available, efficient, and non-stigmatizing low-intensity treatments [10]. Our monthly surveys over a full year and latent-class analyses mitigate the problem that most research has been unable to track the effects of constantly and rapidly changing work environment and accumulation of adversity to subpopulations of employees, thus complementing empiric understanding on COVID-19 related prolonged exposures and mental health of healthcare workers.

Many healthcare workers may have experienced a double burden of stress from COVID-19 compared to the general population, given their similar or higher risk to own health and the additional work stress from treating surges of patients with an infectious disease [11]. It has been shown that workload due to infections-related hospital ward overcrowding may lead to depression and sickness absence in healthcare staff [12–14]. Several targeted meta-analyses suggest that healthcare workers, especially female nursing staff with a close contact to COVID-19 patients, may be at higher risk of psychiatric symptoms during the COVID-19 pandemic compared to other professionals [[3, 15–17], but see [4]].

Various mental-health trajectories during the COVID-19 pandemic have been observed amongst the general population, depending on factors like local lockdowns, personal financial difficulties, pre-existing conditions, and contracting the infection [18, 19]. As for potential traumatic events in general [20], there are inevitable individual differences in individual and social resources to cope with stress from COVID-19, as well as differences between local microenvironments such as wards or departments. While some employees face extreme stress from pandemic work and unemployment in family, others may face primarily stress reductions e.g. from not having to physically travel to work. As such (typically) unobserved factors stay more constant in time than the rapidly varying observed factors like infection incidence, one might expect latent clusters in the healthcare workers’ response to prolonged COVID-19 pandemic over time. Also differential phenotypic human stress responses may give rise to latent clusters of mental-health trajectories following traumatic events [20]. Modeling latent clusters amounts to capturing such between-individual differences in individual-level temporal variation.

Although some studies have monitored healthcare workers mental health during the COVID-19 pandemic either at frequent intervals or over long periods of time (e.g., [1, 21, 22]), few, if any, have done both. Both are needed, however, to accurately detect temporal changes in effects of exposures and for accurate modeling of differential employee trajectories in mental health. This paper examines factors affecting healthcare workers’ mental-health trajectories over time during the COVID-19 pandemic using monthly personnel follow up across a full year of pandemic conditions.

The entirety of the personnel of the HUS Helsinki University Hospital (*n* = 25,494) was invited to participate in a baseline online survey during a difficult early phase of COVID-19 pandemic in June 2020, with 4804 employees (19%) answering [23]. These employees then received monthly follow-up surveys for over a year, with focus here being on how their mental health changed rather than on the baseline representativeness for all of HUS. Until a half-year follow up, we found that temporal variations in local COVID-19 incidence rates correlated with the personnel’s psychological distress. Frontline work and pandemic-related potentially traumatic events (PTE) further increased the risk of psychological distress [24]. Nursing staff were more likely to be at the frontline, but the half-year follow-up data did not allow us to determine whether this explained their greater risk for experiencing psychological distress. Furthermore, as vaccination coverage has increased and the pandemic continued, one might anticipate less clear a link between local COVID-19 incidence and employees’ psychological distress and increasing heterogeneity in how the employees cope with the pandemic in their private lives.

We followed psychological distress and the symptoms of insomnia in the HUS hospital personnel over one year during the COVID-19 pandemic, also modeling risk factors of and latent heterogeneity (clusters) in psychological distress. First, it is important to know how the overall mental health of healthcare workers changes upon prolongation of a pandemic e.g. because accumulating stressful working conditions may reduce quality of care and lead to sickness absences [25–27] and because much of the work on COVID-19 has focused on cross-sectional or sparsely sampled longitudinal samples. Our monthly follow ups better track the rapidly changing local epidemic situation while still covering long exposure times. Second, similar tracking of mild insomnia symptoms in each wave seems important, as they could become a feasible early intervention target for stress mitigation [e.g. [10]]. Third, as latent cluster analysis allows stratification with respect to unobserved covariates (and unknown factors), examining latent clusters in healthcare workers’ mental-health trajectories during the prolonging pandemic may provide important information regarding what employers and occupational health service providers are currently missing, as well as general knowledge on individual differences in mental-health response to the prolonged COVID-19 pandemic. Evidence exists for latent heterogeneity (clusters) in the general population [18, 19], but this study maps their extent in the presumably more homogeneous sub-population of healthcare workers under COVID-19 pandemic.

## Methods

### Setting and participants

The HUS Helsinki University Hospital serves the 26 municipalities in the region of Uusimaa in Finland, a population of 1.7 million. The region includes the capital city of Helsinki, many smaller cities, and sub-urban and rural areas. Before COVID-19 and subsequent economic and political turbulence, in 2019, the human development index of Uusimaa region was 0.958 [28], which is considered very high (top-ranking country, Norway, had the value 0.957 in 2019 on this United Nation’s composite index of life expectancy, education, and income indicators). HUS provides state-funded specialized healthcare to the entire region. The target population of this study were the healthcare works of HUS, who were electronically surveyed.

This study was approved by the HUS Ethical Committee. Permission to conduct the study was obtained from the HUS Joint Resources. After our initial publications on the baseline data and the half-year follow up [23, 24], this paper more thoroughly investigates temporal trajectories over monthly follow-up data spanning an entire year—the period from 4th June 2020 to 28th May 2021, totaling 358 days. The initial sample (*n* = 4804) contained nursing staff (62%), medical doctors (9%), special personnel such as psychologists and social workers (8%), and other non-healthcare personnel (21%), and overall predominantly (88%) females [23]. Average age was 44 years (s.d. 11 years), with a broad range of educational backgrounds: Bachelor’s degree equivalent was the most common highest educational level (55%), but also many doctoral-degree equivalents (10%) were in the sample (see [23] for a full breakdown). Although a bit over half the sample (53%) was lost to attrition in 1st follow-up survey, most returned to at least some of the 11 follow-up surveys (Table 1 shows the number of respondents and other data by each survey wave).

**Table 1:** Average sample characteristics by wave

	Survey wave (waves 0-6 in year 2020, waves 7-11 in year 2021)											
Variable	0	1	2	3	4	5	6	7	8	9	10	11
Date of 1st answer per wave (mm:dd) ^a^	06:04	07:03	08:07	09:04	10:02	11:06	12:04	01:08	02:05	03:05	04:09	05:07
N	4804	2262	2172	1923	1913	1744	1685	1598	1579	1578	1523	1364
At least 1 re-participation (%) ^b^	–	46.82	63.18	69.19	73.11	75.65	77.54	78.48	79.23	80.14	81.12	81.72
Woman (%) ^c^	88.60	89.33	89.58	88.82	89.03	88.86	89.29	88.85	88.60	88.26	88.28	88.74
Age (years)	46.74	49.55	48.50	48.18	48.89	48.31	49.54	47.68	47.78	49.63	48.08	47.86
Direct care (%) ^d^	24.40	14.32	14.19	14.19	16.83	16.76	20.27	19.35	19.30	20.11	19.47	18.43
Nursing staff (%) ^e^	63.03	62.30	61.02	59.85	59.64	60.71	61.54	58.99	58.87	58.69	58.80	60.18
Work changes (%) ^f^	82.37	39.48	29.31	37.05	40.20	23.04	38.97	20.72	18.01	29.88	24.81	17.13
MHI-5 (score) ^g^	72.32	78.25	78.80	76.49	74.98	74.42	71.69	74.78	74.67	72.86	74.52	75.79
MHI-5 ≤ 52 (%)	16.66	9.41	9.05	12.02	14.24	15.02	19.14	13.59	14.25	17.06	13.52	13.45
ISI (score) ^h^	7.11	6.33	6.05	6.35	6.60	6.61	6.95	6.86	6.52	6.64	6.59	6.42
ISI ≥ 15 (%)	9.53	5.88	5.57	7.54	7.58	7.51	8.78	8.76	8.23	8.43	8.40	7.55
PTE (%) ^i^	27.85	18.60	15.89	13.23	13.61	12.81	17.18	14.95	11.76	15.99	14.84	13.24
PTE1 (%) ^j^	12.99	8.28	6.26	5.21	4.74	4.41	6.38	5.00	3.68	6.32	5.87	5.03
PTE2 (%) ^k^	19.87	12.00	9.74	8.35	8.76	7.74	11.74	9.63	7.43	9.63	7.25	5.92
PTE3 (%) ^l^	2.84	2.92	3.37	2.57	2.81	3.03	3.31	3.56	3.51	4.36	4.77	4.80
PTE4 (%) ^m^	0.83	1.12	1.12	1.00	1.11	0.87	0.84	1.40	1.21	1.86	1.49	1.50

^a^ Month and date (format mm:dd) of the first recorded answer per wave. The year-part of the datum is 2020 for the waves 0-6 and 2021 for the waves 7-11

^b^ At least one re-participation to surveys after the zero survey wave

^c^ Woman or someone not indentifying as man

^d^ Directly caring for COVID-19 patients

^e^ Belonging to nursing staff at baseline (i.e., in survey wave 0)

^f^ Reporting changes in work due to COVID-19

^g^ MHI-5 (Mental Health Index −5 rating, 0-100 points, under 53 refer to psychological distress)

^h^ ISI (Insomnia severity index rating 0-28 points, 15 or over refer to moderate or severe insomnia symptoms)

^i^ Potentially traumatic event (any of PTE 1-4, questions i-l below)

^j^ Has your work with suspected or confirmed COVID-19 patients included exceptionally disturbing or distressing assignments?

^k^ Have you had strong anxiety due to your own or close one’s risk of contracting serious illness for your work with suspected or confirmed COVID-19 patients?

^l^ Have you or your close one contracted a severe COVID-19 that required hospital care?

^m^ Has a close one to you died of COVID-19?

### Procedures

An e-mail invitation pertaining to an electronic survey delivered both in Finnish and in Swedish languages was sent to all 25,494 employees of HUS. Besides this initial invitation, an open-access link was available in the personnel’s internal website to capture additional employees undergoing work changes or turnover. The survey consisted of demographic background questions, five symptom rating scales (two used here; see below), and questions pertaining to changes in daily work, adjustment to those changes, attitudes towards COVID-19 patients, and open questions on possible need for psychological support. The survey took about ten to fifteen minutes to answer. The 4804 employees (19% of the HUS personnel) who participated in the initial June 2020 electronic survey, have received invitations to participate also in the monthly follow-up rounds. Although the pressure of dealing with COVID-19 likely limited representativeness of the initial cross-sectional sample, altogether 82% of participants were at least partly retained in follow ups.

In addition to the employee-level survey data dated by the time of responding, weekly COVID-19 incidence rates in Uusimaa region were drawn from an open data repository of the Finnish Institute of Health and Welfare (https://sampo.thl.fi/pivot/prod/en/epirapo/covid19case/). Hence, prevailing incidence rate could be matched to persons’ responses with a one week accuracy.

### Measures

We assessed mental-health problems with the (additive inverse of) the five-item Mental Health Index (MHI-5), which is the mental-health subscale of the RAND-36 (SF-36) self-report questionnaire of health-related quality of life [29]. MHI-5 rating scale consists of five questions: “how much of the time during the last month have you” (1) been a very nervous person, (2) felt downhearted and blue, (3) felt calm and peaceful, (4) felt so down in the dumps that nothing could cheer you up, and (5) been a happy person? There is a six-point Likert-response scale, with reverse scoring for the items 3 and 5 asking about positive feelings. All item scores were then converted to a score between values 0 to 100 [29], with low scores indicating more psychological distress. As in previous Finnish studies, we defined clinically significant psychological distress by MHI-5 scores 52 or below [30–33]. We used Insomnia Severity Index (ISI) to assess sleep problems. ISI is a reliable and valid instrument to quantify perceived insomnia severity [34, 35]. It can serve as a screening device and we applied ISI scores of 15 or over to indicate presence of sleep problems [35]. Regarding statistical modeling, we considered as outcome variables MHI-5 score as a continuous-valued variable, psychological distress (MHI-5 ≤ 52), and high ISI (≥15).

Besides the abovementioned validated measures, we used the following simple tailored questions on potentially traumatic events related to COVID-19: (1) Has your work with suspected or confirmed COVID-19 patients included exceptionally disturbing or distressing assignments; (2) have you had strong anxiety due to your own or close one’s risk of contracting serious illness for your work with suspected or confirmed COVID-19 patients; (3) have you or your close one contracted a severe COVID-19 that required hospital care; and (4) has a close one to you died of COVID-19? If any of 1-4 was endorsed, the employee was considered as having experienced a COVID-19 related PTE. In addition to the PTE questions, we asked whether the employees’ daily work had changed due to COVID-19, whether they had directly provided care to a patient with confirmed or suspected COVID-19 infection during the last week, whether they were members of the nursing staff, and their age and gender (see previous publications regarding questionnaire items and scales not used here, [23, 24]).

### Statistical analyses

We drew boxplots and local regression lines to illustrate the data to the reader. We then modeled the associations between risk factors and time-varying outcomes with multilevel (mixed-effects) logistic regression models, using a random intercept to model employee-specific risks that are constant over the repeated monthly measurements [36, 37]. All the data points (survey answers at given time) with all model-required variables available were used in modeling despite the employee lacking some survey waves. That is, the multilevel models take an approach wherein each study participant contributes to model estimates in proportion to study waves they provide a full data for. In this sense, the multilevel model was “made to solve” the problem of missing values in the outcome variable [e.g., [38], section 7.3.3]. Participants having only part of the predictor variables for one wave do not contribute to that wave, however. In case of MHI-5 outcome, 9.7% of all observations with the outcome available lacked other model-relevant data. For ISI, the predictor-related attrition was 10.1%. These observations were removed during modeling because multiple model structures were investigated and the methods to impute multilevel and latent class structures are presently at immature phase [38]. The actual realized sample sizes per model are given as part of the results tables for both observations and persons in the model.

To understand the overall risk of psychological distress or sleep problems, multiple regression models were fitted with different combinations of covariates in order to adjust their effects on each other in a controlled manner. The effects of calendar time in these models were taken in account via two routes: with polynomials of standardized calendar dates and/or with time-specific local COVID-19 log-incidence per week. More specifically, we strived to first exhaust the effects of simple calendar time by adding polynomials of time until a non-significant higher order polynomial was encountered. Furthermore, we transformed the incidence rate defined as weekly number of new cases *r* to log(*r* + 1) for regression modeling to linearize the generally exponential growth rate of infection transmission.

To understand possible latent classes (clusters) of risk, we modeled such latent heterogeneity in mental health trajectories using latent class mixed models [39]. In an analogy to the overall-risk modeling, we first selected a degree for polynomial expansion that maximized Bayesian information criterion for a single-class model. In a previous research on the general population of the UK [18], even such complexity-adjusted statistical fit criteria tended to suggest very many latent classes, and therefore in that study the number of classes was cut from the first major drop in the relative Bayesian information criterion values instead of seeking for an absolute minimum. We also looked at that criterion and found that the ensuing number of classes was also favored by integrated complete likelihood criterion that combines Bayesian information criterion with entropy-based classification accuracy [40, 41]. In our case, only these criteria maintained reasonably dissociable latent classes (< 30% posterior confusion probability for class membership in each class; see Supplementary Material for additional details) and were thus used. Regression predictions of latent-class memberships were estimated as a part of a single model rather than in multiple steps (i.e., without extracting each employees’ membership) to ensure correct propagation of uncertainty [39]. We used in the analyses R version 4.0.2 (2020-06-22) and lme4 (version 1.1-23) and the lcmm (version 1.9.3) packages [37, 39].

## Results

### Sample characteristics

**Fig. 1:**
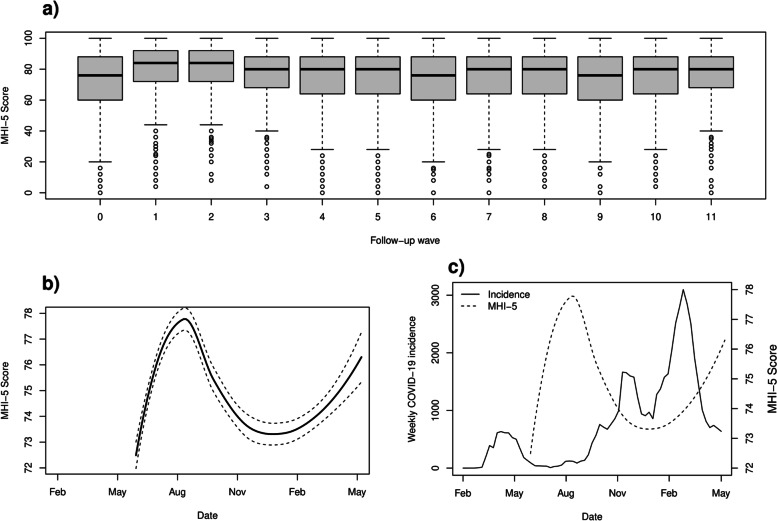
A 1-year monthly follow up of employee mental health plus local COVID-19 incidence. **a)** Boxplot of the 5-item Mental Health Index (MHI-5) by survey wave. **b)** Local regression estimate of MHI-5 score on exact response date (solid line) with 95% confidence intervals (dashed lines; note: these data start from June 2020, i.e., later than the official incidence data). **c)** Weekly incidence of COVID-19 locally (in Uusimaa region, total population 1.7 million). Solid line and left y-axis gives the incidence rates, whereas the dashed line and right y-axis re-state the time-smoothed average MHI-5 score from panel c for illustration purposes. Note: these incidence data are shown from beginning of the pandemic record up to our last survey response from the 12th survey wave #11). The incidence data are from the official open-access record by National Institute of Health and Welfare in Finland

The survey waves approximately reflect the time the participants answered the online questionnaire, with some variation within the waves. Besides large variation between individuals (Fig. 1a), a mean-level pattern across the survey waves was evident for mental health (Fig. 1b), such that it roughly inverse-tracked the local progression of COVID-19 case rate (Fig. 1c). On average, the mental health index was low right after the first peak of the epidemic, increased during the subsequent relatively serene phase of the epidemic, and deteriorated again with the re-emergence of infections (Fig. 1c).

### Correlates of psychological distress and sleep problems during a full year of pandemic conditions

Table 2 shows a set of multilevel models predicting clinically significant psychological distress (i.e., MHI-5 score ≤ 52). A fifth degree polynomial of time was sufficient to capture the time trends of psychological distress over the year of COVID-19 pandemic when incidence was not explicitly modeled (Model 1, Table 2). From the Model 1, we further observed that being a member of the nursing staff and having the COVID-19 contact independently increased the risk of psychological distress. Being a nurse did not moderate the effect of the COVID-19 contact, however (*p* = 0.105 for interaction). The differential risk of psychological distress between professions did not withstand adjusting for PTEs (Model 2, Table 2), meaning PTEs may explain it. Yet, nurses on average were not statistically significantly more prone to PTEs compared to the others (OR = 1.82, 95% CI = 0.84–3.99, when adjusting age, sex, and employee-specific random intercept).

**Table 2:** Multilevel models predicting psychological distress

	Model 1 (N_obs_ = 22,300; N_per_ = 4400)		Model 2 (N_obs_ = 21,670; N_per_ = 4366)		Model 3 (N_obs_ = 21,670; N_per_ = 4366)		Model 4 (N_obs_ = 21,670; N_per_ = 4366)		Model 5 (N_obs_ = 21,670; N_per_ = 4366)	
Fixed effects	OR	CI	OR	CI	OR	CI	OR	CI	OR	CI
z(age)	0.58	0.51–0.67	0.63	0.55–0.71	0.62	0.55–0.7	0.62	0.55–0.71	0.62	0.55–0.70
Sex (female)	2.02	1.28–3.19	1.88	1.25–2.83	1.88	1.25–2.82	1.88	1.25–2.83	1.87	1.24–2.81
Direct care	1.56	1.29–1.88	1.33	1.11–1.61	1.42	1.18–1.71	1.33	1.1–1.6	1.38	1.15–1.66
Nursing staff	1.35	1.00–1.83	1.09	0.84–1.43	1.06	0.81–1.39	1.09	0.83–1.43	1.08	0.82–1.41
PTE	–	–	4.59	3.86–5.45	4.81	4.06–5.71	4.57	3.85–5.43	4.61	3.88–5.47
log(r + 1)^*^	–	–	–	–	1.26	1.21–1.32	1.21	1.02–1.43	1.54	1.42–1.68
z(time)	2.65	2.31–3.03	2.58	2.25–2.95	–	–	1.62	1.06–2.49	0.40	0.25–0.64
z(time)^2^	0.19	0.14–0.26	0.20	0.14–0.27	–	–	0.30	0.18–0.49	–	–
z(time)^4^	2.81	2.36–3.34	2.61	2.19–3.10	–	–	2.10	1.63–2.71	–	–
z(time)^5^	0.67	0.63–0.71	0.70	0.66–0.74	–	–	0.77	0.7–0.85	–	–
log(r + 1) × z(time)	–	–	–	–	–	–	–	–	1.10	1.03–1.18
Random effect										
σ_B_	–	17.39	–	10.72	–	10.28	–	10.81	–	10.42

OR = odds ratio; CI = confidence interval; “–” = variable not in the model; Nobs = number of obser-vations, or person-waves, modeled; Nper = number of persons modeled); z(time) = Questionnaire answering time-variable standardized (z-score transformed) to mean 0 and variance 1; z(time)^*k*^ = *k*^th^ polynomial of the time variable; σ_B_ = Random-effect variance for between-employee differences in risk of psychological distress; A × B = interaction effect for A-by-B; "Direct care" = delivering direct care to COVID-19 patients

^*^ Logarithm of weekly COVID-19 incidence, plus one case to prevent minus infinite log-values

We then investigated the associations between COVID-19 incidence and psychological distress in healthcare workers. Despite both being fixed across individuals, the regional COVID-19 incidence was a partly independent risk factor from the polynomial expansion of calendar time (Models 3 vs 4 in Table 2). This suggests either temporally evolving mental-health impact of COVID-19 incidence or the emergence of other time-dependent processes affecting psychological distress than COVID-19 incidence. To test whether a linear time trend interacted with mental-health impact of COVID-19 incidence, we additionally introduced main effect of standardized calendar time and its interaction with incidence to Model 3 of Table 2 (Model 5; note: Model 4 did not converge with the interaction effect). This increased the OR of log-incidence from 1.26 (95% CI 1.21–1.32) to 1.54 (CI = 1.42–1.68), with the effect of log-incidence increasing by time (interaction’s OR = 1.10, CI = 1.03–1.18) and with calendar time itself having a risk-reducing effect (OR = 0.4, CI = 0.25–0.64). Given these and other evidence for temporally evolving mental-health effect of local incidence, we turned to latent-class modeling (in below sub-section) to detect possible other time-dependent heterogeneity in mental-health trajectories during pandemic.

Sleep problems were studied as an outcome distinct to psychological distress. A COVID-19 contact also increased risk of sleep problems, whereas being a nurse did not (Model 1, Table 3). Relative to results on psychological distress, sleep problems were more strongly associated with provision of direct care to COVID-19 patients and less strongly with PTEs and local COVID-19 incidence (Table 3). However, qualitatively [pertaining to direction of association] the findings for sleep problems were rather similar to those for psychological distress (Table 3 vs. 2). Temporally stable between-individual differences contributed more to risk of sleep problems than to risk of psychological distress (Table 3 vs. 2 random-effect variance), and sleep problems lacked dissociable effects for time vs. COVID-19 incidence (Table 3, Model 3 vs 4).

**Table 3:** Multilevel models predicting sleep problems

	Model 1		Model 2		Model 3		Model 4		Model 5	
	(*N*_obs_ = 22,342; *N*_per_ = 4402)		(*N*_obs_ = 21,710; *N*_per_ = 4368)		(*N*_obs_ = 21,710; *N*_per_ = 4368)		(*N*_obs_ = 21,710; *N*_per_ = 4368)		(*N*_obs_ = 21,710; *N*_per_ = 4368)	
Fixed effects	OR	CI	OR	CI	OR	CI	OR	CI	OR	CI
z(age)	0.84	0.69–1.03	0.87	0.71–1.06	0.86	0.70–1.05	0.87	0.71–1.06	0.86	0.71–1.05
Sex (female)	1.52	0.75–3.08	1.45	0.72–2.93	1.46	0.72–2.95	1.45	0.72–2.93	1.46	0.72–2.94
Direct care	1.66	1.28–2.16	1.48	1.14–1.93	1.62	1.25–2.10	1.49	1.14–1.94	1.59	1.22–2.07
Nursing staff	1.07	0.69–1.67	0.99	0.64–1.54	0.96	0.62–1.50	0.99	0.64–1.54	0.97	0.62–1.51
PTE	–	–	2.52	1.99–3.20	2.71	2.14–3.42	2.53	1.99–3.20	2.63	2.07–3.32
log(r + 1)	–	–	–	–	1.14	1.08–1.21	0.88	0.70–1.10	1.25	1.11–1.40
z(time)	1.97	1.64–2.36	2.02	1.68–2.43	–	–	2.81	1.55–5.08	0.47	0.24–0.89
z(time)^2^	0.39	0.25–0.6	0.36	0.23–0.57	–	–	0.27	0.14–0.53	–	–
z(time)^4^	2.04	1.61–2.58	2.05	1.61–2.61	–	–	2.39	1.68–3.42	–	–
z(time)^5^	0.75	0.69–0.81	0.75	0.69–0.81	–	–	0.70	0.61–0.81	–	–
log(r + 1) × z(time)	–	–	–	–	–	–	–	–	1.11	1.01–1.23
Random effect										
σ_B_	–	62.86	–	57.94	–	57.25	–	57.95	–	57.22

OR = odds ratio; CI = confidence interval; “–” = variable not in the model; *N*_obs_ = number of observations, or person-waves, modeled; *N*_per_ = number of persons modeled); z(time) = Questionnaire answering time-variable standardized (z-score transformed) to mean 0 and variance 1; z(time)^*k*^ = *k*^th^ polynomial of the time variable; σ_B_ = Random-effect variance for between-employee differences in risk of psychological distress; A × B = interaction effect for A-by-B; "Direct care" = delivering direct care to COVID-19 patients

^*^ Logarithm of weekly COVID-19 incidence, plus one case to prevent minus infinite log-values

### Latent classes in mental-health trajectories during a prolonged pandemic

**Fig. 2:**
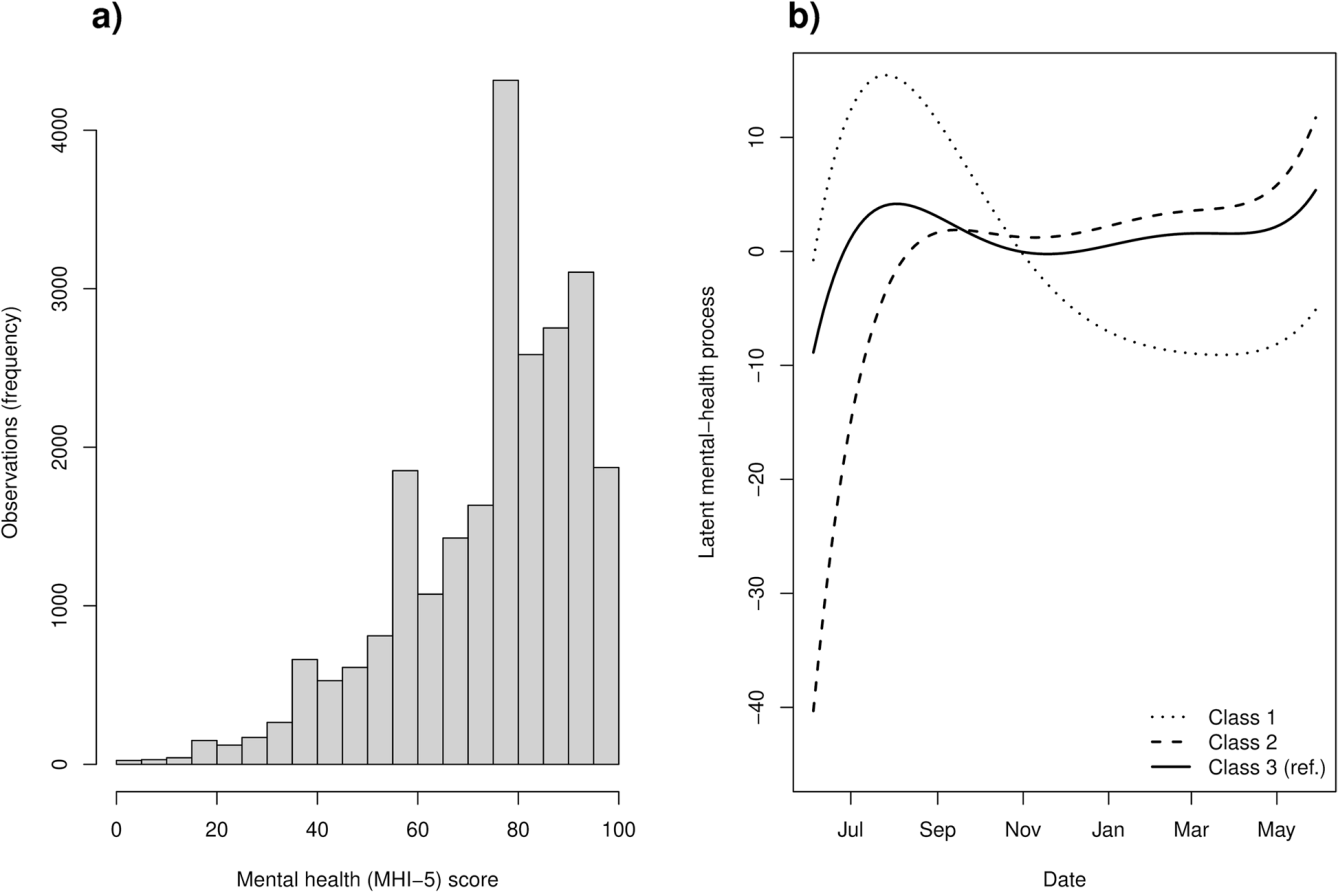
Latent class mixed modeling (lcmm). **a)** Distribution of observations of mental health (MHI-5) scores is supported strictly on interval from 0 to 100 and negatively skewed, suggesting re-scaled Beta distribution for the lcmm link function. **b)** Estimated latent trajectories from a 3-class lcmm model predicting MHI-5 scores with a degree 5 polynomial expansion of time, plus age and sex. The class memberships were further modeled with specific potentially traumatic events and living alone (Table 3; see Supplementary Material for a full set of numeric estimates). We named the latent classes “early resilience, deteriorating” (class 1), “early shock, improving” (class 2), and “stable mental health” (class 3, reference class)

We estimated three latent classes to underlie the observed trajectories of continuous-valued MHI-5 scores in the sample (see Methods and Supplementary Material). As the MHI-5 score was heavily left-skewed with a clear upper limit (Fig. 2a), we linked it with a latent normal variate using a rescaled cumulative Beta distribution [39]. Figure 2b shows the average latent-class trajectories in these latent normal-variate units, with higher values implying better mental health. All the classes showed a rapid initial improvement of mental health after the shock from the first peak epidemic, but otherwise their trajectories differed from each other.

The employees in the third (reference) latent class (62% of all employees) showed relatively stable trajectories with less fluctuation compared to the other classes (Fig. 2b, solid line; we named this class “stable mental health”). The employees in the second latent class (14%) suffered the most from the initial shock but adjusted the best with the prolonged pandemic conditions (Fig. 2b, dashed line; “early shock, improving”). The employees in the first latent class (24%) seemed to suffer less than the others from the initial shock but more than the others from prolonged pandemic conditions (Fig. 2b, dotted line; “early resilience, deteriorating”).

Age, sex, delivering direct COVID-19 care, nursing profession, or COVID-19 incidence were not associated with membership of these latent classes, however—only PTEs were. Some of the specific PTEs we assessed pertained work life, whereas others pertained infection transmission risk in private life (see Table 4 covariate descriptions). As risk of transmission ought to vary by living arrangements, we strived to unpack the meaning of the observed three latent classes by regressing them on both the specific PTEs and living arrangement (living alone) within the original latent-class mixed model (joint estimation). The employees in the class “early shock, improving” (class 2) were the most likely to live alone and the most likely to have experienced a PTE compared to the other groups (Table 4; see Supplementary Material for the full joint set of model parameters).

**Table 4:** Multinomial regression coefficients from the class-membership part of a latent-class mixed model (see Supplementary Material for the entire model)

	Class 1 (ref. Class 3)		Class 2 (ref. Class 3)	
Fixed effects on class membership	OR	CI	OR	CI
PTE 1: work with COVID-19 patients shocking or burdening	2.69	1.51–4.81	12.79	7.02–23.33
PTE 2: own or close one’s risk of severe illness provoked severe anxiety	16.96	7.12–40.42	80.09	30.72–208.84
PTE 3: self or close one hospitalized for COVID-19 infection	2.33	0.86–6.28	1.82	0.51–6.47
PTE 4: relative or close one passed away due to COVID-19 infection	0.39	0.06–2.38	4.29	0.62–29.75
Living alone	1.87	1.34–2.62	3.19	1.90–5.38

*Note: Odds ratios (OR) are for class membership predictions, with 95% Wald confidence intervals (CI) given in the adjacent columns. The reference class 3 (“stable mental health”) was estimated to contain 62% (n = 2704) of employees in the available sample, whereas the class 1 (“early resilience, deteriorating”) contained 24% (n = 1040) and class 2 (“early shock, improving”) contained 14% (n = 614) of employees*

In a supplementary analysis, we modified the Model 2 of Table 2 to show that living alone predicted psychological distress after adjusting for PTEs and that the influence of PTEs on mental health decreased over time. The second latent class could have captured some of these effects. Accordingly, Supplementary Material point estimates (Table S3 for full breakdown) indicated that 92% of employees in the class “early shock, improving” experienced a PTE at baseline and 36% in the last follow-up. In contrast, 6% of employees in the reference class “stable mental health” experienced a PTE in baseline and 7% in the last follow up. Across all the waves, the latent classes 1 (“early resilience, deteriorating”), 2 (“early shock, improving”), and 3 (“stable mental health”) on average had 25.9, 52.5, and 6.2% employees with a PTE, respectively, and 28.2, 33.0, and 16.9% employees who lived alone. Occasionally, the latent groups had very large prevalence differences, which explains the high OR-values in Table 4 (e.g., 79.2% vs. 0.8% prevalence for PTE2, “own or close one’s risk of severe illness provoked severe anxiety”, in classes 2 vs. 3).

## Discussion

In this one-year follow up of hospital employees, both nursing profession and frontline pandemic work increased risk of psychological distress amongst healthcare workers. This long follow up also revealed a dissociation of COVID-19 incidence and calendar time as risk factors for psychological distress, but not as risk factors for insomnia. As in previous reports, COVID-19-related PTEs remained an important risk factor for psychological distress but the impact of the PTEs declined in time, whereas the impact of COVID-19 incidence increased in time. Our results were broadly in line with a Canadian follow-up study [1] but with a larger sample, monthly follow-ups, and modeling of latent classes. Mental health over the prolonging pandemic period reflected three latent classes, differentially associated with PTEs and living conditions. Each resembled one of four primary PTE response trajectories identified in a recent review [20]. We next discuss the findings in the light of the three study questions—prolonging pandemics and (1) psychological distress and (2) sleep problems, and (3) trajectory heterogeneity amongst healthcare workers.

### Prolonging pandemic, pandemic work, and psychological distress

When we in June 2020 first initiated follow up of Finnish hospital employees in HUS region [23], 43.4% of the employees directly involved in COVID-19 pandemic patient care reported potentially traumatic COVID-19-related events—a proportion much greater than for personnel not in direct COVID-19 patient care (21.8%). While some indexes derived from social media have suggested a return to a new normal after first pandemic wave, with associated decline in anxiety [42], our follow up surveys half a year later revealed that also the later epidemic waves hit hard on the employee and population mental well-being in Finland [24]. As elsewhere, we observed frontline nursing staff to be at higher risk than others, but in the half-year data, the effect of frontline work could not be differentiated from the effect of being a member of nursing staff [24]. Here we observed that both nursing profession and frontline work carried partly independent risks. The former effect did not withstand adjusting for PTEs, suggesting that nurses may either experience more frequent COVID-19 pandemic-related events or be more affected by their negative experiences compared to other healthcare professionals.

Irrespective of specific work assignments, increases in COVID-19 incidence strained the healthcare system, possibly implying higher work load or more difficult work to the employees, which could lead to subsequent work stress and psychological distress. Such adverse effects on mental health could alleviate as the system and its employees adjusts to high COVID-19 incidence, but they could also increase due to stress prolongation or moral injuries (symptoms resulting from being unable to provide known-to-be-good care and thereby violating core moral beliefs) [11]. Indeed, we found both that high contemporary incidence increased the risk for psychological distress and that this effect was partly independent of time (i.e., COVID-19 incidence had an independent effect on psychological distress even after adjusting for calendar time). Our finding that the effect of COVID-19 incidence on psychological distress seemed to increase rather than decrease in time appears to speak against the “adjusting to situation” –hypothesis. We return to other explanations upon comparing the mental-health findings to sleep problems in below.

In all our models, experiencing a PTE consistently associated with four- to five-fold risk of psychological distress, but these experiences did not distribute uniformly in time. Although Finland was less affected by the first COVID-19 waves than many other European countries [43], health care workers faced situations which could induce moral injury [44], and there was shortage of personal protective equipment in the early phase of the pandemic which may have increased the fear of getting the infection and infecting others. In addition, the novelty of the threat in itself may have evoked anxiety. Accordingly, we observed the greatest rates of reported PTEs amongst the personnel during the first survey waves, and PTEs had greatest effect on psychological distress at that time (Table 1 and Supplementary Material Table S1). These are merely population-mean trends, however. The pandemic does not treat all people the same, but its psychological consequences are associated with individual differences e.g. in personal resources and social and demographic factors [18, 45]. We return to PTEs in our discussion on heterogeneity of mental health trajectories in below.

### Prolonging pandemic and sleep problems

We examined both psychological distress and sleep problems as outcomes of pandemic working conditions. Sleep problems are a common first psychiatric symptom in prolonged stress and reciprocal relations between insomnia and perceived work stress have been previously established [46]. More generally, unmanageable stress may lead to insomnia via hyper-arousal, whereas fragmented sleep may exacerbate daytime distress via emotional dysregulation [47]. The association between COVID-19-related stress and sleep disturbance among health care personnel and the general population has been shown in earlier studies [48, 49]. Non-COVID-19 studies suggest that low-intensity, mass-deployable treatments for insomnia might generally alleviate common mental health problems [10, 50]. But do sleep problems react to prolonged COVID-19-related stress similarly to psychological distress or do these outcomes part ways as the pandemic conditions continue?

Here, we found both similarities and differences for sleep problems and psychological distress in the hospital employees. Both treating COVID-19 patients and experiencing PTEs were risk factors for both psychological distress and sleep problems, but treating COVID-19 patients had an effect of a comparable magnitude on these outcomes whereas PTEs had almost two times higher effect on psychological distress compared to sleep problems. Furthermore, COVID-19 incidence had an independent effect on psychological distress even after adjusting for calendar time, whereas it did not have such an effect on sleep problems. Notably, the local incidence varied in time but was the same for all the employees. The same was true for the fifth degree polynomial expansion of time already in the model. Thus, the observation of partial independence between incidence and time in predicting psychological distress specifically suggests that novel kinds of incidence effects on psychological distress, but not on sleep, emerged over time. Why might direct patient burden have more prominent time-evolving influence on psychological distress than on sleep problems? Whereas earlier COVID-19 studies have highlighted sleep problems [4, 5] and moral injuries in healthcare workers [11, 44], during our full year of pandemic conditions, we may already begin to see downstream mental-health effects of these early indicators of prolonged stress. As the pandemic prolongs, transient stress reactions versus accumulating adversity may increasingly predict differential health outcomes.

### Latent heterogeneity in one-year mental-health trajectories

We also found evidence for the existence of latent sub-groups amongst the healthcare workers, or latent classes, in which the average trajectories through the pandemic differed across the sub-groups. Employees in the class “early shock, improving” (class 2 in Fig. 2b) experienced great psychological distress during the first survey waves but recovered and stayed at the higher mental-health level thereafter. This minority group thus appeared rattled but not affected in the long term in what comes to mental health. Membership in this latent group was predicted particularly by having experienced work with COVID-19 patients shocking or burdening or having severe anxiety related to own or close one’s risk of severe illness. It may be that these people had experienced moral distress [51] and may also have suffered from the lack of protective equipment in the early phases of the pandemic, and may have lacked social support due to living alone. The initial distress experienced by this group alleviated when the treatment protocols of COVID-19 patients improved, shortage of protective equipment alleviated and COVID-19 vaccinations became available.

The majority of the employees followed a steadier mental-health trajectory throughout the pandemic (class 3 of Fig. 2b: “Stable mental health”), but another minority sub-group appeared to be relatively well at the beginning but suffered from the prolonged pandemic (class 1 of Fig. 2b: “early resilience, deteriorating”). This sub-group could contain employees experiencing stronger double-burden with a situation of a family struggling to cope with the pandemic conditions of the surrounding society [18], or individuals whose everyday life and activities were affected by the pandemic restrictions [45]. They may also represent a group coping well with acute stress and emergency work re-arrangements, but gradually decompensating with the prolonged extra workload and responsibility. Increased quantitative and qualitative personnel demands, with shattered work teams, abrupt transfers of personnel, restricted vacations, and challenges to learn, adapt and teach new practices may pose long term stressors with deficient options for recovery. Such conditions may also expose healthcare personnel to moral injuries due to not being able to provide treatment and attention the patients need [11, 52]. When interpreting these groups in terms of the COVID-19 pandemic, however, one should keep in mind that they may as well, or perhaps at the same time, reflect general phenotypic trajectories of human mental-health response to PTEs [20]. Our “stable mental health” group resembled the “resilience” cluster detected in a recent review of such general trajectories, while the “early shock, improving” group resembled “recovery” cluster, and the “early resilience, deteriorating” resembled “delayed onset” of mental-health problems [20].

Further research on how healthcare employees’ and their families have been affected by the pandemic seems important for ensuring societal resilience to such conditions. Work efficacy of employees might significantly suffer from having to worry about how to manage financially or otherwise if a spouse has lost a job or the usual day-care arrangements have been compromised [53]. Financial difficulties have emerged as a risk factor of mental-health problems during the COVID-19 pandemic in previous studies of the UK general population [18, 19]. In our study, living alone was a risk factor for psychological distress but also predicted membership in the latent class where mental health improved from the initial shock. This pattern may pertain to the dual role of loneliness as a factor weakening general mental-health resilience on one hand [54–56] and as an obvious factor protecting from anxiety-provoking viral transmission risk and from risk of household re-arrangements related to COVID-19.

In our previous half-year follow-up paper [24], the data restricted our ability to adjust the exposure of direct COVID-19-care for belonging to nursing staff because the nursing staff had most of the early COVID-19 patient contacts. In the Model 1 of this longer follow up, we observed that both being a member of the nursing staff and having the COVID-19 contact independently increased the risk of psychological distress. In other words, profession may be a protective factor against COVID-19 contacts but not against the consequences of a contact. A COVID-19 contact also increased risk of sleep problems, whereas being a member of the nursing staff did not. The differential mental-health risk between professions did not withstand adjusting for potentially traumatic events, however (Model 2, Table 3), although nursing staff on average was not statistically significantly more prone to such events (OR = 1.82, 95% CI = 0.84–3.99, when adjusting age, sex, and employee-specific random intercept).

### Strengths and limitations

Findings of this study should be interpreted in the light of its limitations. First, the response rate to the baseline survey was relatively low (19%), which could pertain to difficulties in reaching employees with an electronic survey in the middle of pandemic work. Our ability to keep this initial sample through the follow up was comparatively better, however. Therein, our relatively dense (monthly) follow-up sampling may have been a strength, as many who did not fill in the first follow-up survey nevertheless did have a chance to participate in some of the later surveys (47% re-participated to the first follow up but 82% to at least one of the follow ups). The published 2020 personnel composition differed from our baseline sample (Table 1) only slightly and to the direction typical in surveys, with an overrepresentation of women (89% in sample vs. 84% in HR report), older employees (average age 46.7 years vs. 43.5 years), and therefore also nurses who are predominantly women (63% vs. 54%) [57].

Second, we had limited data on socioeconomic background, living conditions, and other individual differences (e.g., personality) that may affect how the employees cope with pandemic working conditions. However, one would expect many of such factors to change slowly relative to stressors during a year of pandemic conditions, and therefore our densely sampling surveys are likely to provide a good overall picture on how the respondents and the employee-population they represent responded to the investigated time-dependent mental-health risk factors.

## Conclusions

As the pandemic conditions exceeded a full year, healthcare workers transient stress reactions as indexed by sleep problems showed both similarities and differences in their correlates compared to general psychological distress. Effects of PTEs and local COVID-19 incidence on psychological distress were visible and changed in time. Furthermore, the mental-health trajectories of the employees under the pandemic year clustered to three distinguishable classes, suggesting latent sources of heterogeneity in how they fared. Hence, while important, meta-analytic work combining cross-sectional estimates in the field risks diluting important sources of mental health. Exposure to direct pandemic patient work, potentially traumatic events related to it, and local burden of transmission may all have influenced employee mental well-being throughout a year of COVID-19 pandemic conditions. However, in addition to controlling such established exposures, continuously following how individual employees cope with the pandemic and assessing need for psychosocial support may be important for both research and human resources if we are unable to rid ourselves from COVID-19 entirely or when future infectious diseases arise.

## Supplementary Information

**Additional file 1.** Supplementary Material. Healthcare workers’ heterogeneous mental-health responses to prolonging Covid-19 pandemic: A full year of monthly follow up in Finland. Supplementary data on a Logistic regression mixed model and on the latent class mixture models.

## Data Availability

The data are not publicly available due to privacy restrictions. Regarding access, please contact the principal investigator Tanja Laukkala (Tanja.Laukkala@hus.fi). Regarding need to access analysis scripts beyond the online Supplementary Material, please contact the corresponding author.
