# Supplementary material for: Healthcare workers’ heterogeneous mental-health responses to prolonging COVID-19 pandemic: a full year of monthly follow up in Finland
Source: BMC Psychiatry. 2022 Nov 19;22:724. doi: 10.1186/s12888-022-04389-x (PMC9675158; doi:10.1186/s12888-022-04389-x)
Supplement: Supplementary file 1 — Additional file 1. Supplementary Material. Healthcare workers’ heterogeneous mental-health responses to prolonging Covid-19 pandemic: A full year of monthly follow up in Finland. Supplementary data on a Logistic regression mixed model and on the latent class mixture models. [file 12888_2022_4389_MOESM1_ESM.pdf]

SUPPLEMENTARY MATERIAL:

Healthcare workers' heterogeneous mental-health responses to prolonging Covid-19 pandemic: A full year of monthly follow up in Finland

Tom Rosenström,[1,*] Katinka Tuisku,[2] Jaana Suvisaari,[3] Eero Pukkala,[4] Kristiina Junttila,[5] Henna Haravuori,[3] Marko Elovainio,[1,6] Toni Haapa,[5] Pekka Jylhä,[2] Tanja Laukkala[2]

[1] Department of Psychology and Logopedics, Faculty of Medicine, University of Helsinki, 00014 Helsinki, Finland

[2] Department of Psychiatry, University of Helsinki and Acute Psychiatry and Consultations, HUS Helsinki University Hospital, 00029 Helsinki, Finland

[3] Finnish Institute for Health and Welfare, Mental Health Team, 00271 Helsinki, Finland

[4] Faculty of Social Sciences, Tampere University, 33100 Tampere, Finland

[5] Nursing Research Center, HUS Helsinki University Hospital and University of Helsinki, 00029 Helsinki, Finland

[6] Finnish Institute for Health and Welfare, Health Services Research, 00271 Helsinki, Finland

[*] Correspondence: *tom.rosenstrom@helsinki.fi*

Contents

Table S1. Logistic regression mixed model predicting low mental health (MHI-5 ≤ 52) in 21587 observations from 4346 individuals (OR = odds ratio; CI = 95% confidence interval). 2

Selection of the latent class mixed model 3

Table S2. Latent-class mixture model-selection summary table. 4

Table S3. Breakdown of variables by survey wave and predicted latent class 5

A summary of the latent class mixed model.. 7

Table S1. Logistic regression mixed model predicting low mental health (MHI-5 ≤ 52) in 21587 observations from 4346 individuals (OR = odds ratio; CI = 95% confidence interval).

**Fixed effects**	**OR**	**CI**
z(age)	0.64	0.56–0.72
Woman	1.88	1.25–2.82
Direct care	1.34	1.11–1.61
Nurse	1.10	0.84–1.44
PTE	4.36	3.67–5.19
Living alone	2.51	1.86–3.38
z(time)	2.90	2.50–3.36
$z(time)^2$	0.19	0.14–0.27
$z(time)^4$	2.58	2.17–3.07
$z(time)^5$	0.70	0.66–0.75
PTE * z(time)	0.69	0.60–0.79
Living alone * z(time)	0.94	0.82–1.07
**Random effect**		
σ_B	–	10.33

Selection of the latent class mixed model

From previous analysis, we knew approximately 5th degree polynomial expansion to be a fitting description of effects of time on the risk of low mental health. This was verified in the latent class mixed model predicting MHI-5 scores through a re-scaled Beta link and just one latent class: relative Bayesian information criteria (ΔBIC) for 4th, 5th, and 6th order expansions being 84.7, 0.0, 5.8, respectively. We then examined relative BIC values for different numbers of latent classes, each having a distinct 5th degree polynomial expansion for time-effects on MHI-5 scores. While absolute BIC minimum appeared elusive, the most dramatic drop in value was over after including three latent classes (see below figure). For large numbers of classes, the class members would begin having a relative high posterior probability of belonging to some other class; in other words, decline in class interpretability might exceed gains in total information when going beyond three classes. Indeed, the three-class solution had the best entropy (classification-based) criterion and the best integrated classification likelihood (ICL) criterion, which combines BIC with a measure of entropy (Table S3). Therefore, we further interpreted a model with three latent classes.

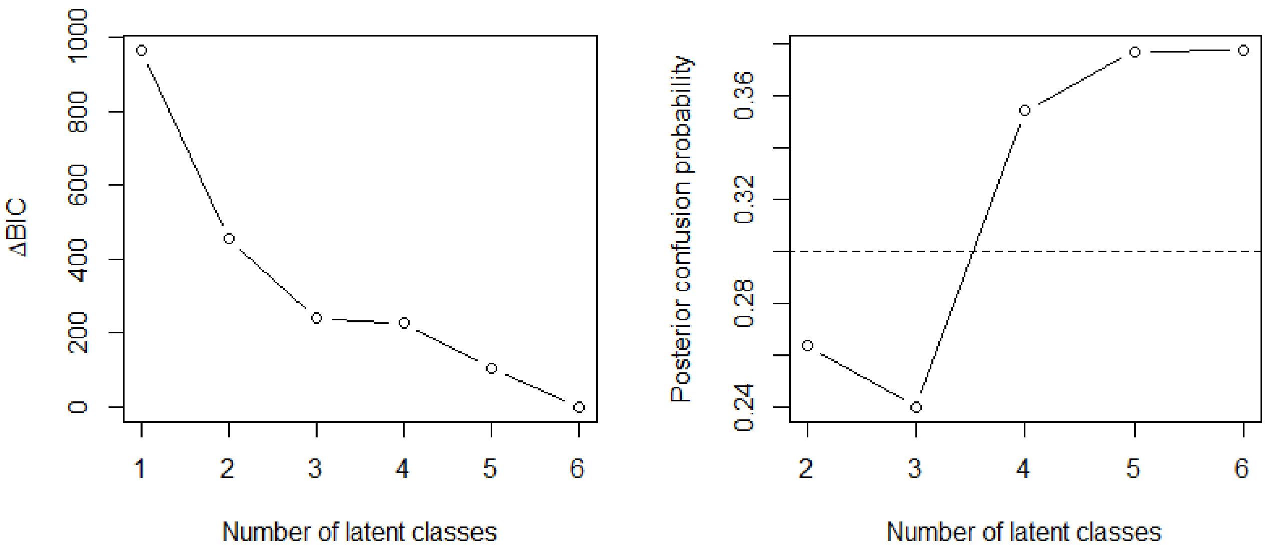

Table S2. Latent-class mixture model-selection summary table.

Number of latent classes	Log-likelihood	Number of parameters	AIC	BIC	SABIC	entropy	ICL
1	-84854.39	12	169732.78	169809.50	169771.37	1.00	169809.50
2	-84569.24	19	169176.48	169297.95	169237.58	0.28	162765.42
3	-84432.28	26	168916.57	169082.80	169000.18	0.61*	161736.80*
4	-84396.47	33	168858.95	169069.94	168965.08	0.49	162729.16
5	-84306.68	40	168693.36	168949.11	168822.01	0.51	162933.30
6	-84224.67*	47	168543.33*	168843.83*	168694.49*	0.57	162555.21

Note: Stars (*) in superscript indicate model favored by the criterion of the column. Lower values are better for Akaike Information Criterion (AIC), Bayesian Information Criterion (BIC), Sample-size Adjusted BIC (SABIC), and Integrated Completed Likelihood (ICL). Higher values are better for log-likelihood and entropy, but entropy is trivially 1 for single-class models.

Table S3. Breakdown of variables by survey wave and predicted latent class

Wave	Class	PTE (%)	PTE1 (%)	PTE2 (%)	PTE3 (%)	PTE4 (%)	Alone (%)	N
0	1	44.72	13.25	33.56	4.79	0.29	29.62	1040
	2	92.12	54.02	79.18	4.59	2.46	33.71	614
	3	6.14	3.51	0.75	1.72	0.64	17.38	2704
1	1	22.29	6.74	14.51	4.02	0.77	30.81	529
	2	57.82	31.74	42.32	7.17	3.41	32.78	299
	3	7.30	3.34	3.34	1.50	0.71	17.57	1286
2	1	21.43	7.08	13.57	4.38	1.03	29.04	489
	2	47.17	23.97	34.21	7.46	2.25	30.15	272
	3	6.70	2.36	2.27	2.26	0.81	17.29	1255
3	1	19.11	5.75	13.02	4.15	0.88	27.39	460
	2	37.56	18.72	28.77	4.48	2.24	32.30	226
	3	5.87	2.23	2.32	1.57	0.83	16.97	1096
4	1	19.95	6.32	13.82	3.70	0.92	27.44	441
	2	41.41	16.67	31.72	6.58	2.19	29.87	231
	3	5.34	1.68	1.96	1.66	0.92	17.06	1102
5	1	17.19	5.17	11.83	3.85	0.78	26.08	395
	2	35.78	14.81	26.48	4.57	1.83	34.70	219
	3	5.82	2.13	1.53	2.42	0.80	15.74	1010
6	1	27.76	7.82	20.05	4.41	0.49	27.64	416
	2	45.27	23.5	35.82	5.47	2.49	29.56	203
	3	6.53	2.28	2.80	2.47	0.64	15.73	947
7	1	23.82	5.79	17.13	5.75	1.09	27.96	372
	2	38.22	18.32	29.32	5.73	3.12	34.69	196
	3	6.55	2.36	2.47	2.22	1.23	16.68	911
8	1	21.2	6.56	15.14	4.95	1.83	29.09	385
	2	32.54	13.02	21.89	7.10	3.55	36.84	171
	3	3.82	0.90	1.35	1.99	0.55	16.76	913
9	1	23.75	9.52	15.57	6.51	1.56	27.51	389
	2	43.62	19.15	31.22	8.47	6.32	35.08	191
	3	6.98	2.67	2.43	2.62	1.02	17.00	888
10	1	22.32	8.36	13.04	5.16	1.43	27.86	359
	2	35.93	17.16	23.08	8.98	5.33	33.53	173
	3	7.11	2.20	1.22	3.70	0.83	17.63	868
11	1	18.71	6.75	10.33	5.45	1.22	24.56	338
	2	35.98	18.29	20.00	8.43	3.61	34.71	170
	3	6.57	1.50	1.22	4.01	1.20	15.82	765

Note: Across all the waves, the latent classes 1, 2, and 3 contained 25.9%, 52.5%, and 6.2% of employees who had potentially traumatic events (PTE), respectively, and 28.2%, 33.0%, and 16.9% of employees who lived alone. Alone refers to living alone (not cohabiting) and the PTEs 1–4 correspond to Table 3 in main text: "work with Covid-19 patients shocking or burdening"; "own or close one's risk of severe illness provoked severe

anxiety"; "self or close one hospitalized for Covid-19 infection"; and "relative or close one passed away due to Covid-19 infection". Predicted class memberships per individual are point estimates subject to uncertainty. Refer to Table 3 in main text regarding uncertainty estimates.

A summary of the latent class mixed model

```
General latent class mixed model
     fitted by maximum likelihood method

lcmm(fixed = mhi5_score ~ zage + sex + poly(zday, degree = 5),
    mixture = ~poly(zday, degree = 5), random = ~1, subject = "IDn",
    classmb = ~pte1 + pte2 + pte3 + pte4 + alone, ng = 3, link = "beta",
    data = dd)

Statistical Model:
     Dataset: dd
     Number of subjects: 4358
     Number of observations: 21590
     Number of observations deleted: 2555
     Number of latent classes: 3
     Number of parameters: 36
     Link function: Standardised Beta CdF

Iteration process:
     Convergence criteria satisfied
     Number of iterations:  8
     Convergence criteria: parameters= 3.3e-05
                         : likelihood= 1.7e-06
                         : second derivatives= 2.7e-09

Goodness-of-fit statistics:
     maximum log-likelihood: -81390.23
     AIC: 162852.47
     BIC: 163082.14


Maximum Likelihood Estimates:

Fixed effects in the class-membership model:
(the class of reference is the last class)

                      coef       Se    Wald p-value
intercept class1  -1.08972  0.14448  -7.542 0.00000
intercept class2  -2.98155  0.24068 -12.388 0.00000
pte1TRUE class1    0.99063  0.29564   3.351 0.00081
pte1TRUE class2    2.54889  0.30646   8.317 0.00000
pte2TRUE class1    2.83092  0.44300   6.390 0.00000
pte2TRUE class2    4.38316  0.48899   8.964 0.00000
pte3TRUE class1    0.84464  0.50645   1.668 0.09536
pte3TRUE class2    0.60006  0.64632   0.928 0.35319
pte4TRUE class1   -0.95336  0.92855  -1.027 0.30455
pte4TRUE class2    1.45563  0.98844   1.473 0.14084
alone class1       0.62670  0.17154   3.653 0.00026
alone class2       1.16109  0.26564   4.371 0.00001

Fixed effects in the longitudinal model:
```

```
                                       coef       Se    Wald p-value
intercept class1 (not estimated)          0                         
intercept class2                   -0.06856  0.19534  -0.351 0.72562
intercept class3                    1.53922  0.12388  12.425 0.00000
zage                                0.26773  0.02243  11.938 0.00000
sex                                -0.24459  0.07226  -3.385 0.00071
poly(zday, degree = 5)1 class1   -77.05140  5.97788 -12.889 0.00000
poly(zday, degree = 5)1 class2    63.79588 10.54028   6.053 0.00000
poly(zday, degree = 5)1 class3     5.46266  2.86162   1.909 0.05627
poly(zday, degree = 5)2 class1    14.91181  3.21908   4.632 0.00000
poly(zday, degree = 5)2 class2   -37.24156  6.80694  -5.471 0.00000
poly(zday, degree = 5)2 class3     0.93754  2.16806   0.432 0.66543
poly(zday, degree = 5)3 class1    32.89727  2.79925  11.752 0.00000
poly(zday, degree = 5)3 class2    43.34485  4.56006   9.505 0.00000
poly(zday, degree = 5)3 class3    13.06152  1.74582   7.482 0.00000
poly(zday, degree = 5)4 class1   -21.83756  2.58497  -8.448 0.00000
poly(zday, degree = 5)4 class2   -21.72008  3.54961  -6.119 0.00000
poly(zday, degree = 5)4 class3   -11.95956  1.65937  -7.207 0.00000
poly(zday, degree = 5)5 class1     9.93868  2.60406   3.817 0.00014
poly(zday, degree = 5)5 class2    15.01942  3.53702   4.246 0.00002
poly(zday, degree = 5)5 class3    10.25819  1.63730   6.265 0.00000


Variance-covariance matrix of the random-effects:
          intercept
intercept   1.56669

Residual standard error (not estimated) = 1

Parameters of the link function:

           coef       Se    Wald p-value
Beta1   0.58579  0.02491  23.517 0.00000
Beta2  -0.22215  0.02875  -7.728 0.00000
Beta3   0.48385  0.01093  44.275 0.00000
Beta4   0.08703  0.00131  66.398 0.00000
```
